# Pollution from drug manufacturing: review and perspectives

**DOI:** 10.1098/rstb.2013.0571

**Published:** 2014-11-19

**Authors:** D. G. Joakim Larsson

**Affiliations:** Institute of Biomedicine, The Sahlgrenska Academy, The University of Gothenburg, Gothenburg, Sweden

**Keywords:** pharmaceuticals, environment, manufacture, risk, management

## Abstract

As long ago as the sixteenth century, Paracelsus recognized that ‘the dose makes the poison’. Indeed, environmental concentrations of pharmaceuticals excreted by humans are limited, most importantly because a defined dose is given to just a fraction of the population. By contrast, recent studies have identified direct emission from drug manufacturing as a source of much higher environmental discharges that, in some cases, greatly exceed toxic threshold concentrations. Because production is concentrated in specific locations, the risks are not linked to usage patterns. Furthermore, as the drugs are not consumed, metabolism in the human body does not reduce concentrations. The environmental risks associated with manufacturing therefore comprise a different, wider set of pharmaceuticals compared with those associated with risks from excretion. Although pollution from manufacturing is less widespread, discharges that promote the development of drug-resistant microorganisms can still have global consequences. Risk management also differs between production and excretion in terms of accountability, incentive creation, legal opportunities, substitution possibilities and costs. Herein, I review studies about industrial emissions of pharmaceuticals and the effects associated with exposure to such effluents. I contrast environmental pollution due to manufacturing with that due to excretion in terms of their risks and management and highlight some recent initiatives.

## 1. Emission studies: including a historical perspective

The rapid development of mass spectrometric analytical methods during the past 25 years has paved the way for studies of pharmaceutical residues in the environment. There were scattered, early indications of discharges from manufacturing being a source of active pharmaceutical ingredients (APIs) in the environment [1–6], but at the time these studies received little attention. The discovery of oestrogens in sewage effluents as a cause of the feminization of fish in the late 1990s sparked an exponentially increased interest in pharmaceuticals in the environment, particularly in the role of excreted drugs. Sewage effluents and receiving rivers were thereafter the prime focus for ecotoxicologists who were interested in pharmaceuticals, and this may have turned attention away from potential alternative sources of pharmaceutical residues. The pharmaceutical industry also argued that significant discharge of APIs from manufacturing is unlikely based on several lines of reasoning, including that the extremely high value of drugs would prevent their release for purely economic reasons [7]. This assumption was later demonstrated to be incorrect (table 1). The discovery of diclofenac residues in cattle carcasses as the cause for the vulture population collapse in India and Pakistan taught us that exposure routes are not always predictable [27].

**Table 1.** Studies on treated industrial effluents, waterways, river sediment, soil and groundwater where pollution with APIs from manufacturing is documented.

country	pharmaceuticals detected	matrices/max. concentration	year	references
China	oxytetracycline—antibiotic	effluent: 1065 mg l^−1^	1988	[6]
India	salicylic acid—anti-inflammatory	effluent: 2270 mg l^−1^	1993	[1]
Denmark	sulfonamide antibiotics and intermediates/metabolites	groundwater: sulfaguanidine 1.6 mg l^−1^	1995	[2]
Germany	phenazone and metabolites	groundwater: phenazone 3.95 µg l^−1^ tap water: phenazone 0.4 µg l^−1^	2002	[3]
Germany	phenazone and metabolites	groundwater: phenazone 2.5 µg l^−1^ tap water: phenazone 0.25 µg l^−1^	2004	[4]
Switzerland	venlafaxine—antidepressant	surface water: 0.8 µg l^−1^	2004	[8]
Norway	bacitracin—antibiotic	effluent: up to 250 kg per discharge	2005	[9]
China	oestrogenic sex steroids	effluent: ethinyloestradiol 51 ng l^−1^	2006	[10]
India	many, including fluoroquinolone antibiotics	effluent: ciprofloxacin 31 mg l^−1^	2007	[11]
China	oxytetracycline—antibiotic	effluent: 19.5 mg l^−1^ surface water: 712 µg l^−1^	2008	[12]
China/Taiwan	many	surface water: diclofenac 27 µg l^−1^	2008	[13]
Croatia	sulfonamide antibiotics	effluent: sulfaguanidine more than 1.1 mg l^−1^	2008	[14]
China	penicillin G and its metabolites	effluent: penilloic acid^a^ 44 mg l^−1^ surface water: penilloic acid^a^ 11.6 mg l^−1^	2008	[15]
China/Taiwan	sulfonamides, NSAIDs and other drugs	effluent: sulfametoxazole 1.34 mg l^−1^; ibuprofen 1.5 mg l^−1^	2009	[16]
India	many, including fluoroquinolone antibiotics	effluent: ciprofloxacin 14 mg l^−1^ groundwater: cetirizine 28 µg l^−1^ surface water: ciprofloxacin 6.5 mg l^−1^	2009	[17]
Switzerland	oseltamivir—antiviral	surface water: 160 ng l^−1^	2010	[18]
USA	narcotic opioids	effluent: metaxalone 3.8 mg l^−1^	2010	[19]
India	fluoroquinolone antibiotics	river sediment: ciprofloxacin 914 mg kg^−1^ organic material	2011	[20]
Korea	lincomycin—antibiotic	effluent: 43.9 mg l^−1^	2011	[21]
Israel	venlafaxine and metabolites	effluent: venlafaxine 11.2 µg l^−1^	2012	[22]
Israel	carbamazepine and venlafaxine	effluent: venlafaxine 11.7 mg l^−1b^	2013	[23]
Pakistan	several antibiotics	surface water: sulfamethoxazole 49 µg l^−1^	2013	[24]
India	fluoroquinolone antibiotics	groundwater: ciprofloxacin 770 ng l^−1^ soil: ciprofloxacin 7.2 µg g^−1^ organic matter	2014	[25]
Spain	venlafaxine	effluent: 2.6 µg l^−1^	2014	[26]

^a^Metabolite. Levels of penillicillin G were in the ng l^−1^ range.

^b^The investigated effluent was not discharged directly, but sent to a local wastewater treatment facility for further treatment.

In 2007, the first in a series of papers was published showing very high emissions of pharmaceuticals from drug manufacturers in Patancheru, near Hyderabad, India [11,17,20]. This area is an important hub for the world's production of bulk drugs and features a very large number of industries congregated in a limited area. The concentrations in the effluent from a treatment plant receiving wastewater from about 90 manufacturing units were, for some pharmaceuticals, greater than those found in the blood of patients taking medicine. The concentration of ciprofloxacin, which is a broad-spectrum antibiotic, was as high as 31 mg l^−1^ [11], which is approximately one million times greater than the levels that are regularly found in treated municipal sewage effluents [28] and toxic to a range of organisms. The estimated total release of ciprofloxacin for 1 day was 44 kg, which is equivalent to Sweden's entire consumption over 5 days, or, expressed in another manner, sufficient to treat everyone in a city with 44 000 inhabitants. These discharges have led to pollution of river sediment [20], surface, ground and drinking water [17] to unprecedented levels, and a recent report also demonstrated contamination of irrigated soils [25]. The research attracted much attention from media, which contributed to increased scientific and societal interest in this exposure route [29,30].

Similar to the observations in India, mg^−1^-levels of oxytetracycline in final, treated effluent and the receiving surface waters were identified at a Chinese factory [6,12], and high concentrations of degradation products from penicillin were observed at another factory [15]. The concentrations of ethinyloestradiol in the treated effluent from yet another Chinese plant was 51 ng l^−1^ [10], which is considerably greater than the concentrations found in sewage effluents and is clearly high enough to disturb reproduction in aquatic vertebrates [31]. Other studies in Asia, including Korea [21], Taiwan [13,16] and Pakistan [24], also demonstrated high concentrations of APIs linked to manufacturing discharges. Additionally, there are reports that identify factories, including formulation sites, in the USA [19] and Europe [9,14] as pollution sources, with concentrations of APIs in treated effluents reaching mg l^−1^. A summary of studies that have reported API discharges from manufacturing sites is presented in table 1. Some studies have analysed selected APIs in streams with mixed input, including both municipal and industrial effluents [13,16,24,26]. Here, only really high pharmaceutical levels can with any certainty be attributed to industrial input. In two exceptional cases, an analysis of the ratio between pro-drug (i.e. a precursor chemical compound present in the medical product) and human metabolites has been elegantly applied to identify industrial discharge of the antiviral drug oseltamivir in the Rhine [18] and of the antidepressant venlafaxine at a sewage treatment plant in Jerusalem [22].

Although much new information has emerged in the past 5 years, the picture of pharmaceutical pollution from manufacturing is still highly fragmentary, and how common large emissions of APIs are remains unknown. To the best of my knowledge, only one study reports exclusively low measured levels (ng l^−1^–low µg l^−1^) in industrial effluent [32]. The study was conceived by a pharmaceutical company at their most highly advanced treatment plant, constructed with the aim of managing and pre-treating the otherwise toxic effluent that was previously sent untreated to a local municipal sewage treatment plant. However, under-reporting is likely because negative findings are generally more difficult to publish. Furthermore, negative findings are truly valuable only if it is clear that the correct drugs were analysed during production using a well-defined analytical protocol. Reports of low levels of APIs in industrial wastewater, municipal wastewater or surface water that receives input from manufacturing sites must be evaluated with these caveats in mind [13,16,32,33]. It is very uncommon for API emissions from manufacturing to be specifically regulated. Accordingly, publicly available, self-reported data from industry [32] or monitoring data from authorities are scarce. There are reports of low effluent concentrations of APIs based on theoretical mass balance calculations that assume a constant discharge rate over time, but without any chemical measurements [34]. Clearly, there is a need for wider monitoring of API emissions from manufacturing worldwide, and it is important to allow the publication of negative findings from adequately designed studies. It is also necessary to acknowledge the possibility of spreading environmental contaminants through solid waste or, for some drugs, possibly even air pollution.

## 2. Effect studies

A number of studies have reported various effects on biota that are associated with exposure to effluents from drug manufacturing (table 2). In some cases, the data are presented in conjunction with exposure concentrations of selected APIs. However, with the exception of a few studies about antibiotic-resistant bacteria in antibiotic-contaminated effluent or sediment, studies that can clearly link an effect to a given API are rare. It is plausible that the occurrence of intersex fish downstream from French steroid factories is due to exposure to discharged steroids [46], but analyses of API residues in the water or fish would provide important additional evidence for this hypothesis. Although APIs are particularly potent chemicals, other compounds present in effluents also have the potential to be toxic [41]. Indeed, the same factory that was releasing antibiotics into the sewer system in Oslo, Norway [9], was recently fined for discharging phosphoric acid, thereby killing fish in the receiving river [51]. One study refers to the closure of more than 60 diosgenin (steroid precursor) factories in China to protect water quality, although it is not clear which compounds in the effluent were considered harmful [52]. Some studies have demonstrated adverse effects in the field [36,46,51] or at effluent dilutions expected in the field [11,44]. In other cases, it is difficult to assess exposure in the environment as potential dilution of the industrial effluent in the recipient is not provided [1,6,10,39]. A few investigations have focused on sublethal effects, such as gene expression and changes in enzyme activities [35,43,48], which may provide information on affected physiological functions, underlying mechanisms and responsible chemicals. Field observations of effects are valuable, but all but one field study deal with bacterial communities (table 2). In some cases, the water in the recipient may actually be too toxic for higher organisms to survive, as in Patancheru [11,44]. An increasing number of studies have described how effluent exposure is linked to bacterial antibiotic resistance [37,38,42,45,47,49,50,53,54]. From an ecological point of view, antibiotic resistance is a sign of resilience or adaptation of the community. Whereas resilience is normally considered positive, from a human health perspective, antibiotic resistance is evidently not [55–57]. Peer reviewed studies that address a possible direct link between industrial discharge and observed human health effects are lacking, although a study by Greenpeace reports that several disorders and diseases are highly over-represented in the pharmaceutical-industry-dominated Patancheru area compared with reference villages [58]. There is one study reporting only negative findings, in which the oral administration of industrial effluent to rats produced no detectable response after 5 days [35]. Taken together, the available data indicate that effluents from drug manufacturing can be highly toxic to different aquatic organisms. While the presence of antibiotics generally agrees well with observed effects on antibiotic resistance, it is less clear what constituents of the often complex effluents are responsible for observed effects on eukaryotic organisms. More field studies, investigating the actual impact of discharges on ecosystems, together with comprehensive chemical characterization, would be particularly valuable.

**Table 2.** Effect studies of controlled exposure experiments using effluents from pharmaceutical manufacture (laboratory) and studies that have characterized the effects of exposure on organisms sampled from environments impacted by discharges from such factories (field). Effects related to effluent exposure on the endpoints listed were demonstrated in all studies, except the one on rats [35]. It is indicated if data on exposure concentrations to APIs are available. ‘(yes)’ refers to API analyses that are performed on final effluent from the treatment plant at another sampling occasion.

country	organisms studied	examples of studied effects	field/laboratory	APIs analysed	year	references
Puerto Rico	planktonic bacteria	taxonomic composition of bacterial communities	field	no	1981	[36]
Puerto Rico	marine amphipods	survival and fecundity	laboratory	no	1983	[5]
Denmark	bacteria	antibiotic resistance	field	no	1998	[37]
Denmark	bacteria	antibiotic resistance	field	no	1999	[38]
China	fish, crustaceans	mortality, behaviour and ventilation	laboratory	no	2002	[39]
China	mice	sperm development	laboratory	no	2007	[40]
India	water fleas, bacteria and plants	immobility, development and bioluminescence	laboratory	yes	2007	[11]
Slovenia	water fleas, bacteria	immobility and bioluminescence	laboratory	no	2007	[41]
China	bacteria	taxonomy, antibiotic resistance and resistance gene abundance	field	yes	2009	[42]
India	fish	gene expression, blood chemistry and enzyme activities	laboratory	yes	2009	[43]
India	frogs, fish	growth, malformations, development, behaviour and survival	laboratory	yes	2009	[44]
China	bacteria	antibiotic resistance and resistance gene abundance	field	yes	2010	[45]
France	fish	plasma vitellogenin and intersex	field	no	2010	[46]
China	bacteria	antibiotic resistance and taxonomy	field	yes	2011	[47]
India	microbial communities	antibiotic resistance gene abundance and taxonomy	field	yes	2011	[20]
India	fish	protein expression and enzyme activities	laboratory	yes	2013	[48]
India	bacteria	antibiotic resistance and bacterial genetics	field	(yes)	2013	[49]
India	rats	gene expression, blood chemistry and weight gain	laboratory	yes	2013	[35]
India	bacteria	antibiotic resistance and bacterial genetics	field	(yes)	2013	[50]
Pakistan	bacteria	antibiotic resistance gene abundance	field	yes	2013	[24]
India	bacteria	antibiotic resistance gene abundance	field	yes	2014	[25]

## 3. Risks

The risks that are associated with environmental discharge from pharmaceutical manufacturing differ in several respects from those that are associated with the excretion of drugs. This is primarily due to differences in exposure levels, as effects thresholds are independent of the contamination source. Concentrations of pharmaceuticals due to excretion in municipal sewage effluents are limited because any drug is only used by a small fraction of the population each day, with the potential exception of during a serious epidemic or pandemic outbreak. Furthermore, in many countries, each person also uses a high volume of water resulting in an initial high dilution of faeces and urine. Additionally, many APIs are relatively efficiently removed during sewage treatment. Consequently, concentrations that exceed 10 µg l^−1^ are rarely observed in treated municipal sewage effluents, in which APIs are commonly detected in ng l^−1^ concentrations. However, in low- or middle-income countries, which may exhibit less water use *per capita* and often have inferior sewage treatment, the levels might be somewhat greater [59]. In contrast to excreted APIs, the great majority of the world's production of a given API might be concentrated in a few factories or even a single site. Although it is expected that only a small fraction of the APIs is lost through discharge during production, it is apparent that the concentrations in effluents from manufacturing can be several orders of magnitude greater than in municipal sewage effluents. Industrial emissions may indeed be the only route for many APIs to reach environmental concentrations that exceed adverse effect thresholds. Production campaigns and washing of reaction tanks, for example, can lead to highly erratic discharges, thus causing peak concentrations that differ significantly from the relatively stable load of pharmaceutical residues entering municipal sewage treatment plants [57,60]. This variation creates challenges for treatment technologies, regulations and monitoring.

Several approaches have been applied to identify which APIs pose the greatest environmental risks; common predictors on the exposure side of the risk equation include usage volumes and/or, as a derivate thereof, predicted exposure concentrations in water or biota [61]. However, usage patterns and metabolism in the human body most likely do not provide any insight into exposure concentrations downstream from manufacturing. Industrial discharge concentrations are *a priori* more difficult to estimate without access to production details, and exposures may be considerably greater at certain times and locations. Taken together, one can expect that manufacturing discharges cause a greater number of APIs to reach adverse concentrations in the environment. In principle, any API that is not readily degraded has the potential to reach adverse exposure concentration in recipients from manufacturing. Regulations and screening efforts should therefore not be restricted to any particular API or class of drugs.

It seems reasonable that usage and excretion contribute to a larger total mass flow of most APIs to the environment than do manufacturing discharges. However, unless an API is highly persistent and can be transported long distances, it is the concentrations in local recipients that matter in terms of ecological risks. A strong accumulation of APIs in biota at sites that are located far from any discharge point has, to the best of my knowledge, not been reported to date. If long-range transport of APIs is restricted, the manufacture of each API at a limited number of sites (in comparison with their usage) therefore would suggest that the exposure to wildlife is also less widespread, but still of high relevance, particularly adjacent to ecologically sensitive ecosystems. However, resistant pathogenic microorganisms need to develop only once at a single site. Then, heavy drug use, insufficient hygiene and extensive travel habits often take care of their spread [55]. Therefore the risks associated with antimicrobial resistance are global, despite the localized nature of the discharges.

The drugs that we use and excrete can affect our local environment, whereas discharge from drug manufacturing mainly contributes to pollution risks in distant locations from the final user. These discharges are less apparent to most of us and may not be perceived as the user's problem, which is likely one reason for the greater concern with local effluent treatment that addresses risks with excreted drugs. Furthermore, because much of the API production is localized in middle-income countries that are undergoing rapid development, it is quite possible that their wastewater infrastructure is, in general, less advanced [62]. The ‘outsourcing’ of pollution, identified as an issue for several sectors in society, clearly poses a moral challenge for the pharmaceutical industry as well [63].

## 4. Risk management

Management options for discharges from manufacturing and excreted drugs differ in several important aspects. The technical solutions for reducing concentrations in effluents partially overlap, although the often-variable composition and toxic nature of industrial effluents provide additional challenges [23,57,64]. In contrast, during manufacturing it may be easier to prevent a given API from being discharged simply because it does not involve hundreds of thousands of point sources that are distributed across the globe. Thus, it is, at least in theory, feasible to install more advanced technical solutions for all relevant sites at a more moderate total cost.

Two products that are completely exchangeable from a clinical point of view (i.e. they contain the same APIs in the same quantities) will result in identical amounts of excreted APIs in urine and faeces, but may be associated with substantially different pollution loads at the production stage. Therefore, substitution with a clinically interchangeable product has the potential to reduce pollution from manufacturing while maintaining the desired therapeutic effects and not increasing the risk of unwanted side effects. The greatest challenge at the moment is that it is exceedingly difficult to ascertain which discharges are associated with a given product and how severe these emissions are [63]. Substitution is not a viable option if the API is produced by only one company, which is most often the case prior to patent expiration, or if a few or even a single manufacturer satisfies the entire market need for a generic API. Substitution with the intent to reduce emissions from excretion either involves substituting one API with another (usually with a similar mode of action), or switching between administration forms, such as pills, gels and patches. These kinds of substitutions are more difficult to apply without jeopardizing patient safety or efficacy to some extent.

Diclofenac, ethinyloestradiol and oestradiol were recently added to the so-called ‘watch list’ within the European Water Framework Directive [65] but there are, to the best of my knowledge, no regulations regarding surface water levels for any API. Therefore, municipal sewage treatment plants are generally not obligated to reduce concentrations of excreted drugs. Similarly, specified industrial discharge limits for APIs are very rare. However, it is possible to define and apply such limits on a local level when licensing a factory, at least in Europe. Although there are opportunities for regulation by local or national authorities, the downside is that it is difficult to influence discharge limits outside one's legal borders. Indeed, most of the APIs used in the European Union (EU) are produced outside the EU. As a global strategy, Sweden has recently proposed that the EU amend the GMP (Good Manufacturing Practice) framework with environmental criteria [57]. Because any company that exports pharmaceuticals to the EU must follow GMP, such criteria have the potential for a wider impact. One of several challenges is how to define industrial discharge limits based on acceptable environmental concentrations. AstraZeneca has recently published a conceptual approach that may be developed further [60]. As the authors recognize, their approach does not encompass antibiotics and risks for promoting resistance, which may require a different assessment [55,66]. It should also be stressed that the environmental risk assessment required in conjunction with the registration of a medicinal product in Europe or the USA does not take into account emissions from manufacturing [67,68]. The US-regulation, however, provides a possibility to consider this route if there is information which suggests that there are ‘unique emissions circumstances’ posing risks to the environment which are not covered by other legislation [68]. For more information on different legal opportunities to regulate pharmaceutical emissions to the environment, see [69].

Ultimately, to ensure safe discharge levels from drug manufacturing, incentives to invest in and operate efficient wastewater technology are essential. The strongest incentives are economic, including legal obligations which can lead to fines or the withdrawal of operation permits. Many actors, including regulatory bodies, companies that purchase bulk drugs, international investment companies, pharmacies and healthcare organizations that buy and distribute the final products, have the potential to influence or create incentives [57]. As an example, the generic substitution system for state-subsidized medicine that is applied in many countries focuses almost solely on reducing cost and provides no or little incentive for companies to invest in ‘green’ technology. To counteract this lack of economic motivation, the Swedish government has recently proposed a revised system in which pollution control during manufacturing is considered when companies compete to obtain product subsidies. Swedish county councils have also started to request monitoring of emissions during manufacturing when procuring medicines. The role of the media should also not be neglected. If the responsible parties and locations where APIs in medicines are produced were well known, the media could more easily apply pressure to improve the situation [63,70].
